# Clinical Lecture at St. John’s Hotel for Invalids

**Published:** 1856-02

**Authors:** J. P Judkins

**Affiliations:** One of the Attending Surgeons. Cincinnati


*Clinical Lecture at St. John's Hotel for Invalids, corner Third and Plum, Cincinnati.* By Prof. J. P JUDKINS, one of the Attending Surgeons.

*Favus Porrigo—Tinea Favosum.*

CINCINNATI, Oct. 17.

We have here, gentlemen, a disease of the scalp—which is interesting to a medical man in several points of view.

Catharine—aged thirteen, pale, thin and of feeble appearance—first noticed the eruption upon her head about four months ago; we can not learn from her the precise form under which it first made its appearance.

At first view of the case, I met with some difficulty in forming a correct diagnosis, for the characteristic feature of the disease was somewhat masked by secondary productions—resulting from inflammation, caused by scratching, or some irritating applications—but by a careful examination of the predominant morbid element, and the absence of those signs which are peculiar to other eruptions of the scalp, I was enabled to pronounce correctly upon its nature. The crust of *favus* differs, essentially, from that of either *eczema* or *impetigo capitis* or *herpes tonsurant.* You observe one spot near the left parietal eminence, which is entirely bald, and where the skin is not inflamed, but is of a pale or natural color. This small spot was once occupied by one of those crusts.

No pediculi are seen, for our patient, since her admission into the hospital, has been subjected to the care of excellent nurses. A slight offensive odor is emitted by the crusts. When these are detached, alopœcia more or less complete, will probably result. Some scattered points are seen, formed by little elevations of a bright yellow color, through which one or more hairs pass.

At its commencement, this disease is not attended by heat, or itching, and hence it is not often noticed until fully developed. Most frequently, it makes its first appearance on the forehead, at the edge of the hair, but soon spreads, and may extend over most of the scalp. It appears in small elevated spots, size of a small pin's head, of a bright yellow sulphur color—one or more hairs traverse it, and where the hair emerges, there is a conical depression. This form and its color from its supposed resemblance to honeycomb, has given to it the name of favus.

As the crust thickens, its pressure upon the sensitive parts beneath produces irritation—heat, and tingling are experienced, followed by scratching, causing cracks or fissures, in which, often large numbers of pediculi are generated, the whole emits an offensive odor, which by some has been compared to that of mice, and by others to the urine of the cat.

"The crusts are hard and dry, and break with a short fracture, exhibiting within a mealy powder, or a paler yellow color than the external surface." The hair involved in the crust begins to change from the commencement of the disease, much of it falls out, and those that remain are thin and pale. If the affection is old, the hair will not be reproduced; and even when it is cured early, the hair scarcely ever regains its normal appearance, being atrophied, and of a whitish yellow color.

Cases are recorded, where all of the scalp, with parts of the neck and forehead, were encased in one large yellow crust, at the edges of which, some small characteristic favi were invariably seen.

*Causes*—The scrofulous constitution is believed by some to be a predisposing cause. Anything that will cause an impoverished condition of the system, as insufficient or bad food, residence in damp and unwholesome localities, or in crowded, dirty and ill-ventilated apartments. Alibert thought that these conditions alone, without contagion, were sufficient to produce the disease. But the majority of dematologists, who believed in the vegetable nature of this disease, contend that it is produced by contagion, a mealy powder or sporules being the media of transmission; but they say that these parisitical fungi, require for their growth a peculiar soil, and this is found in individuals of a peculiar cachectic condition.

Neligan has seen numerous instances of the propagation of favus by direct contract, from child to child, and from children to adults. Divergie also gives analogous cases, where several persons were affected from wearing the same cap. Cazenave relates one case, of its occurring spontaneously upon the scrotum of a man.

The *diagnosis* of this affection is comparatively easy. No other disease presents the peculiar sulphur-yellow crusts of favus; and there is no secretion or desquamation, as in other forms of eruptions upon the scalp. At its commencement, it might be mistaken for *impetigo;* but the speedy development of pustules in the latter, will readily distinguish it. In difficulty, the microscope would give great assistance. *Prognosis* is only unfavorable from the tenacity of the disease, and the atrophy and loss of hair which it induces. Some writers think that it has a deteriorating effect upon the mind of the patient.

*Seat and Nature of Favus.*—Erasmus Wilson pronounces it a disease of the hair-follicles. Cazenave says that it is a morbid secretion of the crypts which open into the pilous canals. Divergie contends that it differs from all other cutaneous diseases by being a true vegetable production.

Shœnlein, of Germany, was the first who detected its vegetable nature, and Robin, of Paris, the celebrated microscopist, has described it minutely, and represented its various parts by accurate plates.

Gruby, Remark, and J. Hughes Bennet, have induced the disease by innoculation.

Gruby innoculated the bark of an oak with the granular matter of a favus crust, and succeeded in producing a morbid production analogous to the disease on the child's head from which the granular matter was taken; this was presented to the members of the French Institute, and Divergie, who was present, says "that its nature could not be mistaken."

This is a strange phenomenon, gentlemen, and, if true, is it not susceptible of improvement? May they not be able to succeed in ingrafting other species of plants upon these crytogamia? If so, the heads of our heroes could be crowned with a wreath of living laurel. But, unfortunately, some difficulty may be encountered in divesting it of the offensive favus odor. Yet this may not be deemed an insuperable objection, for some of the laurels upon the heads of our so-called great men do not exhale the most delicious perfume in the world. But pardon the digression. Erasmus Wilson, to whom we are indebted for the best work in the English language, upon cutaneous disease, denies the contagion of favus, and also its vegetable nature. He reasons well, and adduces many facts in support of his arguments. He shows the analogy between the cellular tissue of vegetables and animals. He says that depressing causes may lower the vitality of nutritive power, so, as in favus, it may assume the lowest form of organization and yet not be vegetable. His reasoning is strong and plausible, and has the adhesion of the celebrated M. Cazenave. But the majority of dermatologists believe firmly in its vegetable nature.

Before alluding to the treatment, I will give you the opinion of Divergie, as contained in his excellent recently published work:

"First, Favus is a disease entirely different from eczema, impetigo, or ptyriasis of the scalp.

Second, This disease, the original cause of which is unknown, is characterized by the developement of a vegetable production around the bulb of the hair, which last is compressed and invaded by it, and a loss of more or less hair results.

Third, It is essentially contagious, and is transmitted by the organized granules, like vegetables.

Fourth, It is mostly a disease of childhood and adolesence.

Fifth, Although most frequently seated upon the head it may be developed on other parts of the body.

Sixth, It never furnishes a product of secretion when left to itself, but may do so if subjected to the influence of irritating ointments.

Seventh, It may get well spontaneously, but the cure thus obtained is only at an advanced age of life, and although cured, the disease still leaves traces of its existence, by atrophy, or loss of hair.

Eighth, It is one of the cutaneous diseases the most refractory to treatment."

*Treatment.*—A great variety of remedies have been used in the treatment of favus. Many writers still believe that the removal of all the hair involved in the crusts is indispensable to the cure. Methods have been used to remove the hair which inflicted great suffering upon the patient—such as cutting the hair close, then covering with some strongly adhesive plaster, and after remaining for several days, was violently jerked off, pulling the hairs out of their follicles. Death has resulted from this cruel mode of treatment.

The celebrated *Calotte*, so much in vogue at one time, and probably still used in some of the European countries, consists of a mixture of rye flour, verdigris, black pepper, rosin and burgundy pitch; this forms a very adhesive mixture. Spread upon strong linen, it was placed upon the scalp, and after remaining for three or four days, it was forcibly removed, bringing the hair with it. Success has attended its use, but doubtless the disease was, in some degree, modified by some of the ingredients contained in the plaster.

The brothers Mahon succeeded in curing many. Part of their cases treatment consisted in the use of depilatory ointments, which contained quick lime. They required eight or ten months to effect a cure.

Plumb advised the removal of hairs, one by one; this would be rather a tedious process. *Cazenave*, of the hospital St. Louis, in Paris, first modifies the temperament of his patient by the use of bitter infusions, iron and cod liver oil. Locally—after cutting the hair, poultices are applied at night, and alkaline washes are used in the morning. To remove crusts, to facilitate the removal of the hair, he uses preparations of quick lime and sulphuret of lime. When the crusts are off, ointments of calomel, etc., are used. He also prescribed emolient, sulphur and alkaline baths during the course of treatment. He generally succeeds in curing the disease in two or three months. *Divergie*, of the same hospital uses nearly the same constitutional treatment as M. Cazenave. Locally—after cutting the hair, he uses poultices, greases the scalp with lard, washes with soap and water, etc., until the crusts are detached—uses ointments ot tanin and preparations of zinc. Every second day he prescribes a sulphur bath. . After proceeding thus for a fortnight, he increases the strength of the ointments, and removes the remaining hair with the forceps.

Erasmus Wilson places his patient under better hygienic conditions—as air, exercise, clothing and washing—gives Tonics, of which he prefers Iron; he also uses alteratives. He orders the favus crusts to be soaked with oil at bed-time, and to be washed with soap or a lotion of carbonate of soda in the morning. As a local application, he uses the ceratum tiglii, 10 to 30 grains to the ounce, or the ung. hydrag. nitratis, diluted one half; or compound sulphur ointment, with some others. The oil silk cap to be worn. He says that the hairs do not act as irritants, and hence their evulsion is not required, yet he believes that the disease is located in the hair follicles.

I will now give you the treatment proposed by Nelegan, and which will be adopted for this case. The general condition of our patient has been much improved since her admission into the wards of the hospital, although but a few days have elapsed. Her diet will continue to be good, but will consist mostly in milk and farinaceous articles of food.

For internal medication—we will put her on Nelegan's prescription of iodide of arsenic:

℞ Arsenici Iodidi, one grain.
Mannæ duræ, six grains.
Mucilage, q. s. Mix et. ft. xij. pills.

Give one morning, noon and night.

A careful watch must be observed while giving this or any other form of arsenic. If any untoward symptom should appear, such as nausea, vertigo, numbness in any part, cramp in the leg, œdœma, etc , we must stop the use of the article for a few days, and then resume it.

Locally—we will cut her hair close, not shave it, and apply flaxseed poultices for ten or twelve hours. Every morning the part will be washed with an alkaline solution, (carbonate of potash, oz, water, Oi.) Then apply an ointment, composed of carbonate potash oz., lard oz., glycerine oz.—spread upon lint and applied to the part diseased. This ointment will be used twice daily—a close-fitting oil-silk cap to be worn. By these means we may succeed in removing the crusts in two or three days. When the crusts are off, will then use the following:

℞ Iodide of Lead, half a drachm.
Lard, one ounce. Mix well.

Spread on lint, and apply morning and evening; but each application preceded by washing with the carbonate of potash solution.

If the ointment should produce too much irritation, we will omit its use for a day or two, and then resume it; still using, however, the alkaline wash.

Nelegan recommends to use the ointment of this strength for about a fortnight, and then to increase its strength a little; and after conducting the treatment in this manner for three or four weeks, to stop the use of all applications, and watch the part for several days. If any sign of favi re-appears, use the ointment of double strength. He insists upon the constant use of the oil-silk cap during the whole course of treatment; and the iodide of arsenic to be continued until we are certain that a cure has been effected.

November 19.

Here is our little patient, gentlemen, whom you have seen from time to time since she has been under treatment.

From a careful inspection of her head, no sign of favi can be detected; upon the spots deprived of hair, we can see some new hair appearing, it is sparse and thin—atrophied. Her general health is much improved.

I think we can say that the cure is complete.—*Cincinnati Med. Obs'r.*

[Note.—Cases of diseased ankle joint, subsequent to injury; varicose ulcers; and others of interest, have been before the class in this Hospital from time to time, some of which will be reported in a future number.]

